# Impact of tiger nut milk as a substitute for cow milk on the rheological, physiochemical, and organoleptic properties of functional ice cream

**DOI:** 10.1007/s13197-024-06125-7

**Published:** 2024-11-13

**Authors:** Hesham A. Ismail, Wafaa M. Salama, Arwa A. Ali, Rezk A. Awad

**Affiliations:** 1 Dairy Science Department, Faculty of Agriculture, New Valley University, El-Kharga, 72511 Egypt; 2 Dairy Research Department, Food Technology Research Institute, Agricultural Research Center, Giza, Egypt; 3 Food Science Department, Faculty of Agriculture, Ain Shams University, Cairo, Egypt

**Keywords:** Cow milk, Tiger nut milk, Ice cream, Physicochemical properties, Rheological characteristics

## Abstract

Tiger nuts are an excellent food choice due to their rich mineral, fiber, and antioxidant content. Tiger nut milk was replaced by 25, 50, 75, and 100% of cow’s milk in the ice cream mixture. The impact of substituting cow milk with tiger nut milk on the physicochemical, rheological, and organoleptic properties of ice cream was examined. Adding tiger nut milk in a combination resulted ice cream mixes with more fiber and antioxidants and a lower protein and ash levels compared to the control (100% cow milk). Ice cream with tiger nut milk was higher in Fe and Zn than that of control. Dynamic viscosity values of ice cream mixes with tiger nut milk were decreased with increasing values of the applied shear rate compared to the control. All functional ice cream formulations with tiger nut milk showed higher specific gravity and melting rate with lower overrun percentages than the control. Regarding taste and flavor, structure, texture, and melting in the mouth, there is no appreciable difference between the T_25_, T_50_, and control treatments. Therefore, cow’s milk can be replaced with tiger nut milk up to 50% in the ice cream industry to raise the nutritional value and functional properties.

**Supplementary Information:**

The online version contains supplementary material available at 10.1007/s13197-024-06125-7.

## Introduction

People of all ages like ice cream because of its cooling properties and because it’s a food made of milk. In addition to being high in macronutrients like fats, carbohydrates, and proteins, ice cream also contains micronutrients such as minerals (calcium) and vitamins (E, D, and A). Nevertheless, it is deficient in natural antioxidants and dietary fiber (Bhat and Bhat 2012). Moreover, some individuals have completely boycotted these dairy products from their diets, including vegetarians and those with lactose or casein allergies. Consumer preference for functional foods has been increasing recently owing to higher levels of natural antioxidants, dietary fiber, minerals, vitamins, natural colorants, and are free of artificial additives. Thus, the concept of functional emerged to raise the nutritive content of food. Foods or dietary ingredients with additional health advantages beyond their normal nutritional value are referred to as functional foods (Diplock et al. 1999). Because consumers interested in eating healthier and more functional foods, manufacturers of ice cream have been using more ingredients with physiological and nutritional qualities, such as natural antioxidants, probiotics, dietary fibers, alternative sweeteners, and certain fruits (Zhao 2007). Using plant-based milk as a source of plant proteins and lipids in ice cream not only incorporates the health benefits of plant compounds, but it also creates new products with distinctive features, such as lactose-free products, that may appeal to consumers and be beneficial (Ghaderi et al. 2021).

Phyto-milk contains unsaturated fats and is cholesterol-free, both of which improve cardiovascular health. It is rapidly digestible and serves as an excellent and economical substitute for animal milk. Tiger nut (*Cyperus esculentus*)-based milk has been developed as an affordable alternative to conventional cow milk. They are one of the underutilized crops, grows as a crop, a weed, or in the wild. Common names for tiger nuts include “earth almond,” “chufa,” and “zula” nuts. A delightful drink known as “Horchata de Chufas,” or tiger nut milk, can be produced using raw, roasted, dried, baked, or roasted tiger nuts. Tiger nuts are high in natural sugars, protein, dietary fiber, and vitamins (E and C) as well as minerals including phosphorus and potassium (Umelo et al. 2014). Their abundance of energy sources (proteins, carbohydrates and fats) and high soluble glucose and oleic acid content make them a healthy choice for people with high cholesterol, coronary heart disease, arteriosclerosis, heavy digestion, flatulence, dysentery, and cancer, particularly colon cancer. They have been shown to benefit diabetics as well as others trying to lower their cholesterol or lose weight. Tiger nuts are a great food choice because of their high fiber content and tasty flavor since tiger nut flavor and texture are similar to those of coconut. Thus, the objective of this study was to evaluate the physicochemical, rheological, and sensory properties of ice cream made using tiger nut milk at different levels as substitute cow milk.

## Materials and methods

### Materials

The source of fully ripe tiger nuts (*Cyperus esculentus* L.) was obtained from Perfumer Khader Al Attar in Cairo. Fresh cow’s milk was supplied by the dairy unit of the Agriculture Faculty, Dairy Science Department, New Valley University, Egypt. Vanilla powder, sugar (sucrose), and fresh cream were bought from the local market in El-Kharga City, New Valley Governorate, Egypt. Carboxy methyl cellulose (CMC) and skim milk powder were obtained from Dairy America TM, USA. The chemical composition of cow milk, tiger nut milk, skim milk powder and fresh cream is reported in Table S1. Every chemical and reagent used was purchased from Sigma-Aldrich Co. (St. Louis, Mo., U.S.A.).

## Methods

### Preparation of the tiger nut milk

Tiger nut milk was prepared according to (Sanful 2009) with some modifications. The foreign and poor-quality tuber nuts that could have an impact on the milk’s flavor and quality were removed before making the tiger nut milk. Using distilled water, the tiger nut tubers were cleaned and rinsed. After that, it was soaked in water for 24 h, with several changes, to allow the fibers to get softer. The tubers were boiled for 30 min in tap water (1.0 kg of tuber was added to 3 L of warm water) and repeatedly mixed using a blender. The milk was then removed from the mash by filtering it through a muslin cloth and to get a fine consistency, it was further strained. After the tiger nut milk was filtered, it was heat treated for 10 min at 90  °C, and later cooled to a temperature of 5 °C. The filtrate was then kept into clean sterilized plastic containers till for further processes and utilization.

### Preparation of ice cream

Control ice cream mix was adjusted to contain 0.2% CMC, 8% fat, 15% sucrose and 12% milk solids non-fat (MSNF), according to the Egyptian Standard (2005). The cow milk was replaced by tiger nut milk in ice cream mixture at levels 25, 50, 75, and 100%. Control treatment was prepared without adding tiger nut milk and the other treatments were supplemented with different levels of tiger nut milk and henceforth refer as T_25_, T_50_, T_75_, and T_100_, respectively (formulas for the mixes are given in Table 1). The needed amounts of skim milk powder were combined with required sucrose and stabilizer (CMC) and added gradually to the liquid ingredients (milk and cream) at 45  °C while being vigorously stirred. The base mixtures were pasteurized for 10 min. at 80  °C, cooled in an ice bath to 5 ± 2  °C, then aged for 24 h at that same temperature. Subsequently, the mixtures were merged with 0.01% vanilla powder and then whipping-freezing in an Italian Taylor-Male, Model 156 ice cream maker (Fig. S1). Before being evaluated, the manufactured ice cream was filled in 100 g cups and kept at − 18  °C in a freezing cabinet for at least 24 h. Three duplicates of each treatment were done.

**Table 1:** Formulations of ice cream mixes with tiger nut milk in different ratios as substitution of cow’s milk (kg/100kg)

Ingredients	Ice cream treatments*					
	Control	T_25_	T_50_	T_25_	T_75_	T_100_
Sucrose	15	15	15	15	15	15
Skim milk powder	5.78	5.78	5.78	5.78	5.78	5.78
Stabilizer (CMC)	0.2	0.2	0.2	0.2	0.2	0.2
Cream	7.73	7.81	7.87	7.81	7.95	8.19
Tiger nut milk	–	17.8	35.57	17.8	53.3	70.83
Cow milk	71.29	53.41	35.58	53.41	17.77	–
Total	100	100	100	100	100	100

This procedure was used three times to make ice cream., where: *Control: 100% cow milk; T_25_: cow milk substituted with 25% Tiger nut milk; T_50_: cow milk substituted with 50% Tiger nut milk; T_75_: cow milk substituted with 75% Tiger nut milk; T_100_: cow milk substituted with 100% Tiger nut milk

### Physiochemical analysis of ice cream

The AOAC (2005) standard methodologies were used to measure the contents of moisture, total solids, protein, fat, ash, and dietary fiber contents. Total solids-(protein + fat + crude fiber + ash)} was the difference used to compute the total carbohydrates content. Refractometer (Abbe Hergestellt in der DDR, Germany) was used to assess total soluble solids (TSS) content at 20 °C; results were reported in Brix. Antioxidant activity of samples was determined as per Lee et al. (2003). The atomic absorption spectrophotometer, NO.3300 (PerkinElmer, US instrument Division Norwalk, CT, USA), was used to measure the level of minerals described by Hankinson (1975). According to Petersen et al. (2000), the Brookfield Engineering Labs DV III ultra-rheometer, Inc. Stoughton, MA, USA was used to evaluate the apparent viscosity (centi poise) of the functional ice cream treatments. The viscometer was operated at different shear rates and the data were directly recorded from instrument after one minute of spindle rotation to ensure a steady reading. The T-D spindle was selected for the measurement.

### Physical properties of ice cream

After being aged for 24 h and frozen-whipped, ice cream samples were examined. The pH and acidity values were measured using the AOAC (2005) standard procedures. The ice cream’s specific gravity at 20 °C, weight per gallon (Kg), and energy value were computed under the assumption that the energy values of fat, protein, and carbohydrates were, respectively, 8.8, 4.3, and 3.9 kcal/g (Arbuckle 1986). Using a thermometer designed specifically for low temperatures, the freezing point was determined. The following equation was used to determine the overrun of ice cream (%) using the methodology described by Arbuckle (1986): Overrun % = (*Weig*ℎ*t of unit mix* − *weig*ℎ*t of equal volume of ice cream*/*Weig*ℎ*t of equal volume of ice cream*) × 100.

### Rheological characteristics of ice milk

A method outlined by Arbuckle (1986) was used to determine the full time of the first drop, and the melting rate was evaluated as mentioned by Segall, and Goff (2002). Methodology of El-Nagar et al. (2002) was used to assess the melting resistance.

### Sensory evaluation of ice cream

After a 24 h hardening period at − 18 °C, ten semi-trained panelists from Department of Dairy Science, Faculty of Agriculture, New Valley University, Egypt examined ice cream samples for organoleptic quality. Ten minutes at room temperature were used to evaluate the sensory attributes. The samples received ratings of 45 for flavor, 30 for body and texture, 15 for melting quality, and 10 for color (Arbuckle 1986). The total acceptability score was calculated as a summation of flavor, body and texture, color, and melting quality scores.

### Statistical analysis

Utilizing SPSS (version 16 SPSS Inc., Chicago, IL, USA), one-way analysis of variance (ANOVA) was performed at the probability (*P* < 0.05) level of significance to analyze the data. Every sample was examined in three copies. Each and every data point was displayed as a triple average ± standard deviation.

## Results and discussion

### Chemical composition of cow and tiger nut milk

Chemical composition of cow and tiger nut milk is present in Table S1. Findings showed that total solids, fat, protein, carbohydrate, ash, and fiber contents were 13.44, 11.31; 3.95, 2.65; 3.83, 2.45; 4.82, 4.98; 0.84, 0.42 and 0.0, 0.81% for cow and tiger nut milk, respectively. Meanwhile, the acidity percent were 0.18, 0.13; and pH values were 6.78, 7.08 for the two milk types, respectively. Cow milk contain high level of total solids, fat, protein, ash and acidity. On the other side, the cow milk had free crude fibers. However, tiger nut milk has high contained of crude fibers and pH value and low acidity percent compared to cow milk. The digestible carbohydrate contents of cow and tiger nut milk were similar with slightly higher from that of tiger nut milk. These outcomes agreed with the findings of Bristone et al. (2015), who reported that their tiger nut milk had carbohydrate values of 5.81% and cow milk 4.56% that were similar to our study, which were 4.98% and 4.82% for two milk types, respectively.

### Physicochemical composition of ice cream mixes

The results of adding tiger nut milk to the ice cream mixture in place of cow’s milk are displayed in Table 2. The total solids, protein and ash levels of all combinations with tiger nut milk were lower when compared to control, and these values considerably declined as the tiger nut milk addition ratio increased. The ash content decreased significantly with increasing volumes of cow’s milk replacement; it was 0.64% for the T_25_ treatment and 0.37% for the T_100_ treatment. The fat contents of ice milk mixes were not significantly affected with adding tiger nut milk into the blend since it was adjusted in the formula, but the slight differences were possibly because of the slight differences in the chemical composition of tiger nut milk. These results agreed with Shalabi (2023), who found that with the increasing levels of substitution of cow’s skim milk for tiger nut milk, the fat content increased significantly from 1.76% for the 100%% cow’s skim milk treatment to 2.64% for the 100%% tiger nut milk treatment. The carbohydrates content also behaved in the same way as the protein and ash contents in the ice cream mixture. Conversely, tiger nut milk added to the mixture resulted in ice cream mixes with more fibers. This is mostly because tiger nut milk has a higher fiber content and less protein and ash (Table S1). These findings were consistent with El-Shenawy et al. (2016) and Shalabi (2023), about ice cream produced with tiger nut milk. According to Owusu and Owusu (2016), dietary fiber is thought to be essential for the best possible digestive health and provides a number of functional benefits that may be useful in the treatment and prevention of a number of diseases, including diabetes and colon cancer. The high content of TSS was observed in the control sample and decreased gradually when increasing the amount of tiger nut milk substituted. The comparatively low moisture content and high sugar content are the causes of the elevated TSS levels. These outcomes lined up with the findings of Ismail et al. (2020) when studying the characteristics of ice cream enriched with Doum syrup and Pomegranate peel. All treatments with tiger nut milk exhibited lower values of energy (kcal/g) than control, which declined with increasing the substitution level. Due to its high fat and sugar content, ice cream raises the risk of obesity, especially pediatric obesity and associated health issues. This is because it contributes to a high consumption of these nutrients (Krystyjan et al. 2015). Due to these worries, there is a greater need for high-fiber, low-calorie foods. As a result, tiger nut ice cream is one of the new foods that fits well with a healthy lifestyle. The effect of tiger nut milk on some properties of ice cream mixes is also shown in Table 2. Titratable acidity values in ice cream formulas tended to drop when tiger nut milk was added. Tiger nut milk has a higher pH and a lower titratable acidity value than cow’s milk, which is why the pH values rose when the amount of milk was added to the blend at a greater rate (see Table S1). Furthermore, as demonstrated by Table 2, ice cream mixes with tiger nut milk had higher specific gravity values than the control group, and this increase increased with the addition of tiger nut milk to the mixture. Among all treatments, T_100_ sample had the highest value of specific gravity. Specific gravity (sp.gr) of mixes varies with varying mix composition. Because weight per gallon values are calculated using the specific gravity values, the data showed that they followed the same trend as specific gravity. Therefore, T_100_ sample has the highest weight per gallon value. The freezing point of ice cream mixes is significantly impacted by the addition of tiger nut milk. The ice milk formula was improved by adding tiger nut milk, which produced mixes with higher freezing points. The freezing point of T_100_ treatment was the highest, whereas the control treatment had the lowest. The soluble ingredients, such as sweetener, have a direct impact on the freezing points of ice cream mixes. The freezing point of a mixture is affected by the solutes’ type, amount, and molecular weight (Marshall et al. 2003). The data is consistent with Awad’s (2007) finding that soluble ingredients like sweeteners have a direct impact on an ice cream mix’s freezing point, whereas the ratio of fat and protein in the mixture has an indirect effect.

**Table 2:** Physicochemical composition of ice cream mixes with tiger nut milk in different ratios as substitution of cow’s milk

Character assessed (g/100 g)	Treatments*				
	Control	T_25_	T_50_	T_75_	T_100_
Total solids (%)	32.89 ± 0.25^a^	31.91 ± 0.33^b^	31.8 ± 0.04^d^	30.67 ± 0.28^c^	30.22 ± 0.21^c^
Fat (%)	7.85 ± 0.13^a^	7.74 ± 0.14^a^	7.62 ± 0.16^a^	7.58 ± 0.24^a^	7.46 ± 0.11^a^
Total protein (%)	4.88 ± 0.31^a^	4.61 ± 0.23^ab^	4.26 ± 0.13^bc^	4.08 ± 0.14^bc^	3.83 ± 0.13^c^
Ash (%)	0.82 ± 0.01^a^	0.64 ± 0.03^b^	0.58 ± 0.08^b^	0.45 ± 0.03^c^	0.37 ± 0.03^c^
Carbohydrate (by difference)	19.26 ± 0.10^a^	18.68 ± 0.17^b^	18.33 ± 0.07^bc^	18.10 ± 0.18^ cd^	17.93 ± 0.14^d^
Fiber (%)	0.08 ± 0.14^e^	0.24 ± 0.01^d^	0.39 ± 0.03^c^	0.46 ± 0.03^b^	0.63 ± 0.03^a^
Total soluble solid (TSS) ^0^Brix	27.27 ± 2.78^a^	25.90 ± 0.20^ab^	24.30 ± 0.99^b^	23.90 ± 0.53^b^	23.83 ± 0.49^b^
Energy value (kcal/g)	165.18 ± 1.47^a^	160.79 ± 2.04^b^	156.86 ± 0.39^c^	154.84 ± 0.56^c^	152.04 ± 1.48^d^
Titratable acidity (%)	0.31 ± 0.04^a^	0.28 ± 0.01^ab^	0.25 ± 0.03^ab^	0.22 ± 0.01^b^	0.21 ± 0.03^b^
pH value	6.49 ± 0.03^b^	6.58 ± 0.14^ab^	6.66 ± 0.03^ab^	6.71 ± 0.23^ab^	6.87 ± 0.06^a^
Specific gravity (w/v)	1.123 ± 0.01^a^	1.126 ± 0.02^a^	1.131 ± 0.01^a^	1.134 ± 0.04^a^	1.136 ± 0.01^a^
Weight per gallon	4.25 ± 0.01^a^	4.26 ± 0.05^a^	4.28 ± 0.02^a^	4.29 ± 0.02^a^	4.30 ± 0.07^a^
Freezing point	− 2.11 ± 0.04^a^	− 2.04 ± 0.10^ab^	− 1.93 ± 0.03^bc^	− 1.84 ± 0.04^ cd^	− 1.77 ± 0.06^d^
Minerals/contents (mg/100g) **					
Calcium (Ca)	145 ± 4.24^a^	138 ± 4.24^a^	129 ± 2.83^b^	127 ± 1.41^b^	119 ± 1.41^c^
Potassium (K)	241 ± 8.49^a^	231 ± 4.24^ab^	228 ± 2.83^b^	225 ± 2.83^b^	222 ± 1.41^b^
Phosphorus (P)	89 ± 4.24^a^	81 ± 4.24^b^	78 ± 2.83^bc^	72 ± 1.41^cd^	68 ± 1.41^d^
Iron (Fe)	0.18 ± 0.03^b^	0.18 ± 0.01^b^	0.19 ± 0.03^b^	0.21 ± 0.03^ab^	0.26 ± 0.01^a^
Zinc (Zn)	0.31 ± 0.04^d^	0.32 ± 0.01^cd^	0.39 ± 0.03^bc^	0.46 ± 0.03^b^	0.54 ± 0.01^a^
Sodium (Na)	51 ± 4.24^a^	49 ± 2.83^a^	44 ± 1.41^ab^	41 ± 2.83^b^	38 ± 1.41^b^
Magnesium (Mg)	224 ± 2.83^a^	222 ± 1.41^a^	214 ± 4.24^b^	208 ± 2.83^b^	199 ± 1.41^c^
Antioxidant activity (%)	48.24 ± 1.46^e^	55.87 ± 0.88^d^	64.55 ± 0.81^c^	72.19 ± 1.30^b^	79.50 ± 1.44^a^

*see Table 1 for details, **Dry weight basis

Mean values ± standard deviation (SD) of three duplicates of each treatment

^a,^^b,c^: means having different superscript letter(s) in each row differs significantly (*P* < 0.05)

### Mineral contents and antioxidant activity of ice cream mixes

Frozen dairy products are the most popular dairy products worldwide due to their nutritional and functional properties. While having excellent nutritional properties, there is still a lack of some mineral components, such as iron and zinc, which play important roles in the human immune system (Lisak Jakopović et al. 2022). Tiger milk extract contain significant amounts of minerals such as calcium, potassium, phosphorus, iron and zinc. As can be seen in Table 2 that mineral contents (Ca, K, and P) of ice cream treatments with tiger nut milk achieved lower and (Fe and Zn) were higher in these minerals than the control. The higher the percentage of replacement with the extract, the greater the content of these minerals. Antioxidant activity values as indicator of antioxidant contents of ice cream mixes with tiger nut milk are presented in Table 2. According to Si-qun et al. (2016), the flavonoids included in tiger nuts provide them high antioxidant properties, making them a useful natural antioxidant source. All treatments with tiger nut milk exhibited higher values of antioxidant activity than control. These findings corroborated those of Shalabi (2023), who discovered that the antioxidant and total phenolic contents of the cold milk treatments similarly increased significantly (*P* < 0.05) when the ratios of tiger nut milk instead of cow’s skim milk increased. As tiger nut milk is used in place of cow’s milk, the antioxidant activity values of the ice cream combination increase, and as the substitution rates are raised, the antioxidant activity of the finished product were increased.

### Rheological characteristics of ice cream mixes

**Fig. 1:**
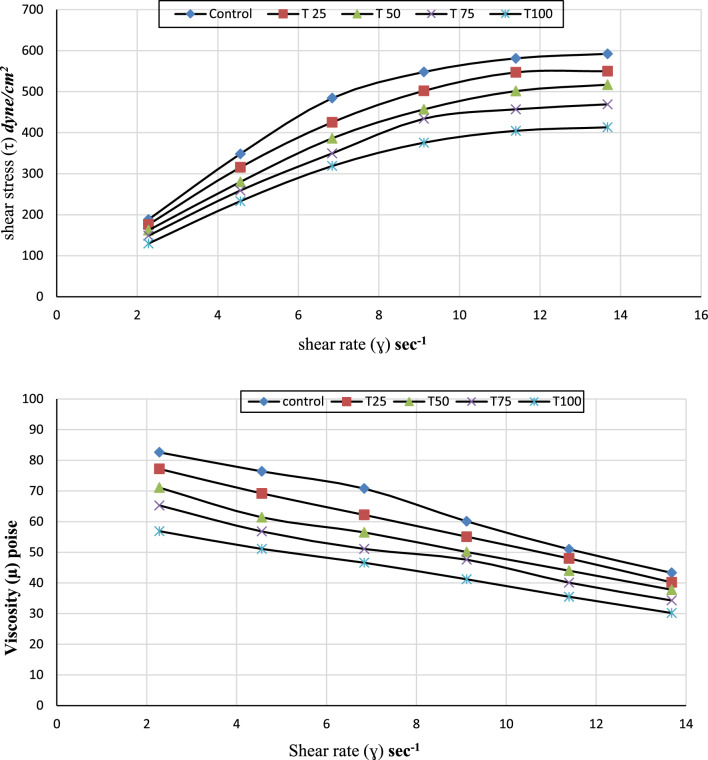
Flow behaviour and Dynamic Viscosity of ice cream mixes with Tiger nut milk in different ratios as substitution of caw milk, where: Control: 100% cow milk; T_25_: cow milk substituted with 25% Tiger nut milk; T_50_: cow milk substituted with 50% Tiger nut milk; T_75_: cow milk substituted with 75% Tiger nut milk; T_100_: cow milk substituted with 100% Tiger nut milk

Ice cream is a frozen foam comprised of partially aggregated lipid particles, air cells, ice crystals, and a continuous aqueous phase (serum) in which components such as polysaccharides, proteins, lactose, and mineral salts are dispersed (Bahramparvar and Mazaheri 2011). Figure 1 displays the shear stress and dynamic viscosity of ice cream mixes made with tiger nut milk substituted in various ratios for cow’s milk. From the data presented it can be seen that control treatment of ice cream mixes showed the highest range of shear stress values (188.25 to 592.21 dynes/cm^2^), while these ranges were decreased by increasing the ratio of substitution to reach lower level of 129.68 to 413.05 dynes/cm^2^at 100% substitutions. Shear stress values of all treatments fortified with tiger nut milk were lower than that of control without addition. This trend was independent of shear rate. The level of shear stress values was decreased in the treatments with increasing the ratio of tiger nut milk substitution. This means that the tiger nut milk is obviously less viscus than cow milk, which in general, decreases the resistance of ice cream mixes to flows and induces shear thinning effect on the rheogram pattern of the mixes. The results are in agreement with Fayed et al. (1996) and Awad and Metwally (2000). Flow behavior rheogram (shear stress/shear rate curves) for ice cream mixes with tiger nut milk as a substitution of cow milk are illustrated in Fig. 1. It represents the pattern of shear stress response with change in the applied shear rates in the range of 2.28 to 13.68 S^−1^. As seen, the course of shear stress response was not linear with the changes in shear rate indicating non-Newtonian behaviour of ice cream mixes. The flow curves were of pseudo-plastic type since the increase in shear stress values ceases with increases in the applied shear rate values. The ice cream mixes exhibited a pseudo-plastic behavior in all treatments including control with existence of a yield stress. Addition of tiger nut milk to the ice cream formula resulted in a downward shifting of the flow curve (destroying of structure leading to decrease in sample viscosity).

For optimal whipping and air cell retention in an ice cream system, a specific degree of viscosity is required (Marshall et al. 2003). The viscosity values of different treatments (Apparent Viscosity µa) were obtained from the rheological data according to the following equation:

1 $$\mu a \, = k \cdot \gamma^{n - 1}$$

where: µa = Apparent viscosity (poise), κ = consistency index (dynes,S^n^/cm^2^), γ = shear rate (S^−1^), and n = flow behaviour index (-).

Ice cream’s viscosity values combine with tiger nut milk as a partial substitution of cow milk in the base formula are presented in Fig. 1. It can be noticed from the data that substitution of cow milk with tiger nut milk in ice cream recipes has significantly affected the apparent viscosity of mixes. In accordance with this, all treated ice cream mixtures’ dynamic viscosity values dropped as the applied shear rate increased. As seen, dynamic viscosity value of control mix sample was 82.6 poise (8260 cp) at low shear rate of 2.8 (S^−1^) and decreased by almost 50% to reach 43.3 poise at shear rate of 13.68 (S^−1^). The data also indicated that control treatment without substitution possessed the highest viscosity values at all shear rates. Increasing the ratio of added tiger nut milk led to lower viscosity of ice cream mixes as shown for T_100_-treatment with 100% tiger nut milk. All substituted mix samples showed similar behaviour, but with lower intensity than that of control sample, so that the dynamic viscosity of 100% tiger nut milk (T_100_) showed dynamic viscosity value of only 30.2 poise at shear rate 13.68 (S^−1^). The variations in the composition of the two milks may be the cause of the variations in viscosity values between the control and treatments with cow milk or tiger nut milk. Because cow milk has a high content of milk protein, which has the potential to become more viscous, it will become more viscous when processed into ice cream mixes. When compared to substituted tiger nut milk, which has a lower amount of plant proteins and a higher amount of water, the higher ratio of milk protein may adsorb and bind a greater amount of free water, enlarging the hydrodynamic volume of the protein molecule, and therefore increasing the viscosity of mix. The findings are consistent with those of Awad and Metwally (2000), who discovered an increase in ice cream viscosity with adding total milk proteinate powder.

### Flow parameters

By using a power law equation to fit the shear stress/shear rate data, the flow behavior of ice cream mixes modified by substituting tiger nut milk for cow milk was assessed (Benezech and Maingonnat 1994) according to the power law pattern of non-Newtonian fluids:

2 $$\tau = k \cdot \gamma ^{n}$$

where: τ = shear stress (dynes/cm^2^).

Power law constants (k and n) for all treatments were obtained using double logarithmic plot (Parnell-Clunies et al. 1986) and given in Table 3. The addition of tiger nut milk in place of cow’s milk did not significantly alter the flow behavior index (n). Based on the flow behavior index, which gauges the degree of deviation from Newtonion flow, the results were consistent with pseudoplastic flow, for which “n” is less than 1.0 (Parnell-Clunies et al. 1986). However, addition of tiger nut milk did not markedly affect “n” values and maintained it around 0.7 showing slight deviation from Newtonion flow. Also, the pseudoplasticity of the flow behaviour was confirmed with the low values of flow behaviour index (n = 0.655 to 0.668). The use of tiger nut milk in ice cream recipes had a greater impact on the consistency coefficient (k) than the flow behavior index (Table 3). Control ice cream sample (100% cow milk) showed the highest value of consistency coefficient, being 121.11 dynes, S^n^/cm^2^, while this value was gradually decreased to the level of 110.52, 98.94, 91.35 and 80.90 dynes, S^n^/cm^2^by increasing the ratio of cow milk replacement by tiger nut milk with 25, 50, 75 and 100%, respectively. The results reveal that there is a direct relationship between the amounts of added tiger nut milk and the k-values. Increasing the amount of added tiger nut milk in ice cream blends resulted in lower k-values as seen in Table 3. The differences in flow parameters of the treatments could be attributed to the different capability of tiger nut milk and cow milk in binding water into the basic matrix. The results are in line with Rohm and Schmid (1993) who studied the flow properties of yoghurt as influenced by dry matter fortification.

**Table 3:** Influence of tiger nut milk substitution of cow milk on yield stress, consistency, flow behaviour index and R^2^of ice cream mixes

Treatments*	Flow behaviour index (n) (-)	Consistency coefficient (k) (Dynes. S^n^/cm^2^)	Yield stress τ) (dyne/cm^2^)	R^2^
Control	0.655	121.1101	73.58	0.9496
T_25_	0.658	110.52039	44.07	0.9669
T_50_	0.668	98.9387	34.64	0.9784
T_75_	0.666	91.3958	31.69	0.9728
T_100_	0.666	80.8989	28.22	0.9673

*see Table 1 for details

The yield stress values are also presented in Table 3 and could be noticed that control sample showed the highest value of 73.58 dyne/cm2 which expresses the gel strength pattern in the ice cream mix. Incorporation of tiger nut milk (25%) as a substitution of cow milk in the mix reduced the yield stress necessary to start the flow of ice cream mix to reach 44.07 dyne/cm^2^. This may be due to the partial disruption of the gel matrix upon addition of tiger nut milk. Increasing the addition level of tiger nut milk to 100% did not remarkably decrease the yield stress value than that of 50% addition because the gel matrix of ice cream mix was already diluted enough at 50% substitution level. Given that the R2 values fell between 0.949 and 0.978, it is evident that the power law model is a good fit for describing the rheological behavior of ice cream mixes.

### Physicochemical properties of resultant ice cream

Table 4 displays some of the characteristics of the final ice cream made with tiger nut milk substituted in various ratios for cow’s milk. The results show that, as compared to the control, the resulting ice cream treatments made with tiger nut milk had higher specific gravity and weight per gallon. This increase was increased by increasing the percentage of replacing cow’s milk with tiger nut milk. On the other hand, the addition of tiger nut milk reduced the overrun percent in ice cream treatments compared to the control. As the amount of tiger nut milk in the final ice cream grew, the overrun decreased more and more. Since the viscosity value of the control was higher than that of any tiger nut milk treatments, the reduced overrun % in ice cream treatments may be connected to the lower viscosity values. Arbuckle (1986) and Marshall et al. (2003) stated that the overrun in ice cream is affected by viscosity values. El-Shenawy et al. (2016) found in a previous study that as the tiger nut extract concentration increased, there was a little decrease in ice cream overrun (*P* > 0.05). Moreover, Shalabi (2023) indicates that the overrun of ice milk treatments significantly decreased (*P* < 0.05) when the variable ratios of tiger nut milk instead of cow’s skim milk grew. Table 4 also, shows the time in minutes required for the first drop of ice cream to which tiger nut milk has been added. The results show that the time required for the first drop was reduced by adding tiger nut milk to the formula. The 100% tiger nut milk treatment (T_100_) took 3.89 min to melt, compared to 9.56 min for the control. The decrease in first drop time could be due to the lower viscosity values of the treatments compared to control as shown in Table 4. The rate of melting rate was taking a trend opposite to the time required for the first drop to fall. Where the melting rate was higher in the treatments than in the control. This was in line with a prior study by El-Shenawy et al. (2016), who found that ice cream prepared with a different ratio of tiger nuts melted more quickly than the control (*P* > 0.05). Table 4 also, displays the melting resistance expressed as a percentage of weight loss from the sample’s starting weight. As the tiger nut milk percentage in the ice cream increased, the melting resistance was dropped, and all functional ice cream formulations with tiger nut milk shown greater melting than control. The variations in the added tiger nut milk percentages and their impact on the mix qualities are the primary cause of the variances in melting resistance across all ice cream treatments. The mixture’s viscosity value has the most effect on the ice milk’s melting resistance. The resistance of ice cream to melt often increases with increasing mix viscosity. According to Arbuckle (1986) the findings are consistent.

**Table 4:** Physicochemical properties of resultant ice cream with tiger nut milk in different ratios as substitution of cow’s milk

Character assessed	Treatments*				
	Control	T_25_	T_50_	T_75_	T_100_
Specific gravity	0.6342 ± 0.02^c^	0.6558 ± 0.01b^c^	0.6789 ± 0.01^ab^	0.7052 ± 0.01^a^	0.7156 ± 0.02^a^
Weight per gallon	2.4004 ± 0.04^b^	2.4822 ± 0.04^ab^	2.5696 ± 0.08^ab^	2.6692 ± 0.05^a^	2.7156 ± 0.17^a^
Overrun	77.11 ± 0.89^a^	72.74 ± 0.14^b^	66.61 ± 0.25^c^	60.86 ± 0.31^d^	58.77 ± 0.18^e^
First drop after (min)	9.56 ± 0.28^a^	9.26 ± 0.11^a^	8.18 ± 0.08^b^	6.32 ± 0.20^c^	3.89 ± 0.14^d^
Melting rate (ml/min)	1.892 ± 0.01^d^	1.979 ± 0.01^ cd^	2.118 ± 0.00^c^	2.337 ± 0.13^b^	2.655 ± 0.03^c^
Melting resistance (%)					
15 min	25.7 ± 0.17^e^	30.5 ± 0.24^d^	34.6 ± 0.17^c^	36.80 ± 0.40^b^	39.80 ± 0.34^a^
30 min	54.70 ± 0.48^e^	55.90 ± 0.17^d^	57.50 ± 0.24^c^	61.40 ± 0.37^b^	64.70 ± 0.42^a^
45 min	73.20 ± 0.45^e^	75.40 ± 0.33^d^	79.70 ± 0.23^c^	83.10 ± 0.49^b^	86. 80 ± 0.28^a^

see Table 1 for details

Mean values ± standard deviation (SD) of three duplicates of each treatment

^a,^^b,c^: means having different superscript letter(s) in each row differs significantly (P < 0.05)

### Sensory evaluation of resultant ice cream

The organoleptic acceptance of ice cream with tiger nut milk was shown in Table 5. Data revealed that the panelists scored all examined ice cream samples with scores shown significant differences from the control sample. When milk from tigers was used instead of cows, all sensory property ratings declined relative to the control, and this effect was amplified when the proportion of replacemen**t **was increased. On the other hand, control treatment scored the highest among all ice cream samples. Treatment T_25_, the ice cream, was rated top out of all the treatments in each quality attributes. There is no discernible difference between the T_25_, T_50_, and control treatments in terms of taste and flavor, body, texture, or melting in the mouth. Substituting more than 50% cow’s milk resulted in a substantial difference between the organoleptic qualities of the T_75_ and T_100_ treatments. When up to 50% tiger nut milk was used, the resulting ice cream generally received extremely high marks for acceptance, texture, and flavor. This means that functional ice cream can be made with 50% tiger nut milk as a substitution for cow’s milk, which close to control in sensory and physical attributes but with higher nutritional quality. These findings mimic those of Shalabi (2023) and El-Shenawy et al. (2016).

**Table 5:** Sensory evaluation of resultant ice cream with tiger nut milk in different ratios as substitution of cow’s milk

Treatments*	Character assessed				
	Flavor (45)	Body & texture (30)	Melting (15)	Colour (10)	Acceptability (100)
Control	43.00 ± 1.80 ^a^	27.00 ± 1.58 ^a^	13.00 ± 0.87 ^a^	9.11 ± 0.60 ^a^	92.11 ± 2.37 ^a^
T_25_	41.91 ± 2.37 ^ab^	26.26 ± 1.94 ^ab^	12.11 ± 1.69 ^a^	8.86 ± 0.53 ^a^	89.14 ± 3.78 ^a^
T_50_	39.44 ± 2.60^bc^	25.78 ± 1.79 ^bc^	11.74 ± 1.59 ^ab^	8.22 ± 1.20^ab^	85.18 ± 5.16 ^ab^
T_75_	35.52 ± 4.30 ^c^	23.67 ± 1.58 ^c^	10.25 ± 1.66 ^b^	7.44 ± 1.41 ^bc^	76.88 ± 6.37 ^bc^
T_100_	31.67 ± 5.00 ^d^	21.89 ± 5.47 ^c^	8.96 ± 2.30 ^c^	6.82 ± 1.39 ^c^	69.34 ± 7.79 ^c^

*see Table 1 for details

Mean values ± standard deviation (SD) of three duplicates of each treatment

^a,^^b,c^: means having different superscript letter(s) in each column differs significantly (*P* < 0.05)

## Conclusion

Plant-based milks are in the spotlight thanks to their lactose-free, animal protein-free, and cholesterol-free features, which fit well with the current demand for healthy food products and which appeal to consumers and be advantageous. Tiger nut milk is a good source of fiber and natural antioxidants, as well as being distinctive in minerals (iron and zinc). Therefore, replacing tiger nut milk with cow’s milk leads to compensate for those nutrients that it lacks and supporting ice cream with them. As well as creating new products with distinctive features, such as lactose-free products, that may appeal to consumers. From the above, it can be concluded that cow’s milk would be replaced with tiger milk up to 50% (T_50_) in the ice cream industry as a source of fibers, minerals, and antioxidants to raise the nutritional value and functional properties of the resulting product. In future work, probiotic ice cream will be manufactured from tiger nut milk and fortified with fruits, for that to be more acceptable and healthier for consumer.

## Supplementary Information

Below is the link to the electronic supplementary material.

Supplementary file1 (DOCX 1594 KB)

## Data Availability

The authors confirm that the data supporting the findings of this study are available within the article and its supplementary materials.
