# Supplementary material for: Impact of tiger nut milk as a substitute for cow milk on the rheological, physiochemical, and organoleptic properties of functional ice cream
Source: J Food Sci Technol. 2024 Nov 13;62(8):1537–46. doi: 10.1007/s13197-024-06125-7 (PMC12214104; doi:10.1007/s13197-024-06125-7)
Supplement: Supplementary file 1 — Supplementary file1 (DOCX 1594 KB) [file 13197_2024_6125_MOESM1_ESM.docx]

**Table S1 Physiochemical properties of the ingredients used in the formulation of ice cream mixes**

| **Ingredients** | **Total solids %** | **Fat %** | **Protein %** | ***Carbo%** | **Ash %** | **Fiber%** | **Acidity** | **pH value** |
| --- | --- | --- | --- | --- | --- | --- | --- | --- |
| **Cow Milk** | 13.44 | 3.95 | 3.83 | 4.82 | 0.84 | 0.0 | 0.18 | 6.78 |
| **Tiger Milk** | 11.31 | 2.65 | 2.45 | 4.98 | 0.42 | 0.81 | 0.13 | 7.08 |
| **Skim milk powder** | 96.1 | 1.69 | 33.96 | 52.27 | 8.18 | 0.0 | 1.12 | 6.61 |
| **Fresh cream** | 57.49 | 53.52 | 1.36 | 2.13 | 0.49 | 0.0 | 0.11 | 6.77 |

Data: Mean values *carbohydrates


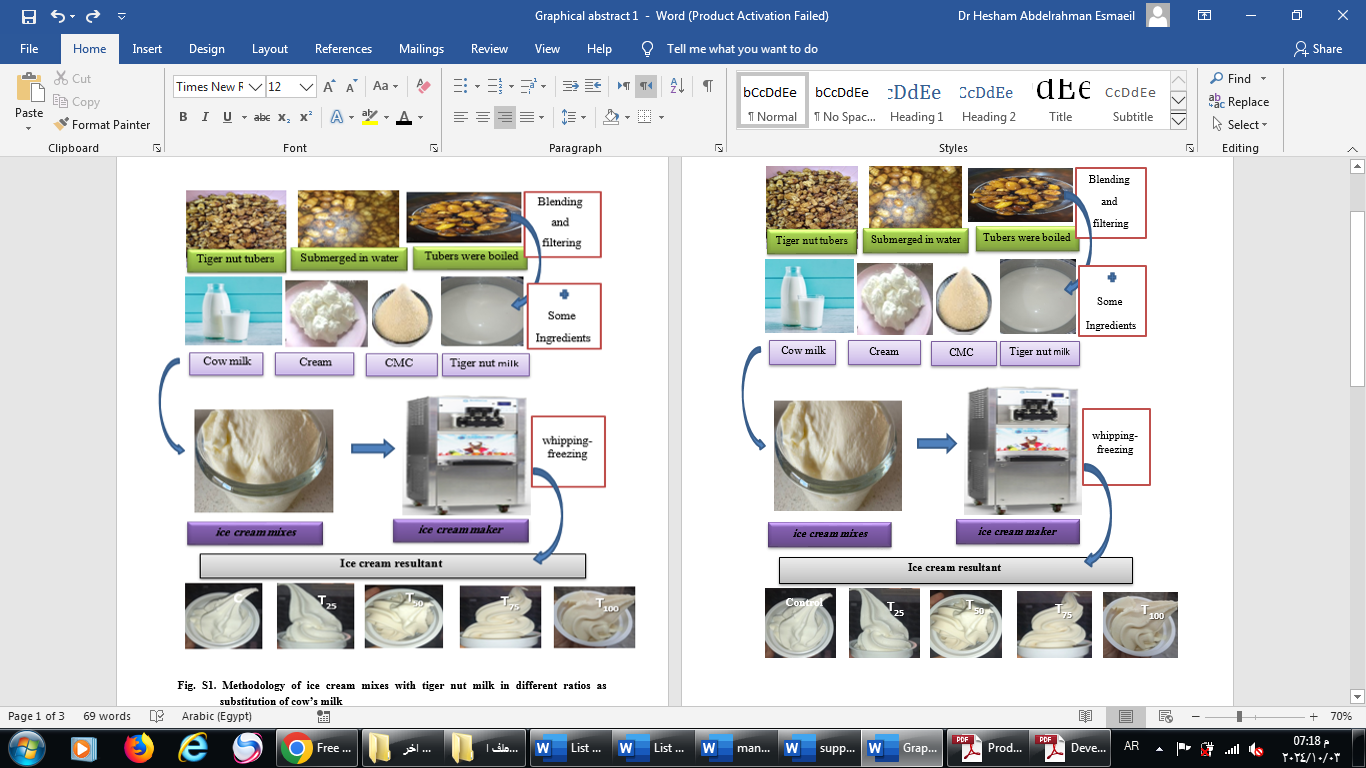


**Soaked in water**

***Ice cream maker***

***Ice cream mixes***

**Fig. S1. Methodology of ice cream made with tiger nut milk in different ratios as substitution of cow’s milk**

Fig S1 Methodology of ice cream with tiger nut milk in different ratios as substitution of cow’s milk.
